# Selenoprotein K Is Essential for the Migration and Phagocytosis of Immature Dendritic Cells

**DOI:** 10.3390/antiox11071264

**Published:** 2022-06-27

**Authors:** Huan Xia, Yongmei Wang, Jie Dai, Xin Zhang, Jun Zhou, Zhu Zeng, Yi Jia

**Affiliations:** 1 Key Laboratory of Infectious Immune and Antibody Engineering of Guizhou Province, Cellular Immunotherapy Engineering Research Center of Guizhou Province, School of Biology and Engineering/School of Basic Medical Sciences, Guizhou Medical University, Guiyang 550025, China; xiahuan@stu.gmc.edu.cn (H.X.); Wangyongmei@stu.gmc.edu.cn (Y.W.); daijie@gmc.edu.cn (J.D.); zhangxin@stu.gmc.edu.cn (X.Z.); 2 Immune Cells and Antibody Engineering Research Center of Guizhou Province, Key Laboratory of Biology and Medical Engineering, Guizhou Medical University, Guiyang 550025, China; 3 Department of Pathology, Guizhou Qiannan People’s Hospital, Qiannan 558000, China; 4 Hubei Key Laboratory of Bioinorganic Chemistry & Materia Medica, Hubei Engineering Research Center for Biomaterials and Medical Protective Materials, Key Laboratory of Material Chemistry for Energy Conversion and Storage, Ministry of Education, School of Chemistry and Chemical Engineering, Huazhong University of Science and Technology, 1037 Luoyu Road, Wuhan 430074, China; 5 Shenzhen Huazhong University of Science and Technology Research Institute, 9 Yuexing Third Road, Shenzhen 518057, China

**Keywords:** selenoprotein K, dendritic cells, endoplasmic reticulum stress

## Abstract

Selenoprotein K (SELENOK) is an endoplasmic reticulum stress (ERS)-regulated protein required for the calcium (Ca^2+^) flux-mediated migration of T cells and neutrophils, and the migration and phagocytosis of macrophages and microglia. However, the effect of SELENOK on the regulation of the immune function of dendritic cells (DCs), including immature DCs (imDCs) and mature DCs (mDCs), is still unclear. In this study, imDCs prepared from SELENOK knockout mice were used to evaluate the effect of SELENOK on the migration and phagocytosis of imDCs. The results showed that ERS-induced downregulation of imDCs phenotypic markers led to a reduction in Ras homolog gene family member A (RhoA)-dependent migration and enhanced Ca^2+^/CD205-mediated phagocytosis. SELENOK deficiency-induced upregulation of selenoprotein S (SELENOS) attenuated ERS levels in imDCs. An increase in Ca^2+^ levels resulted in increased migration and decreased phagocytosis with or without ERS conditions. The migration was RhoA-dependent, and Ca^2+^ or CD205 was associated with regulating phagocytosis in imDCs. Our study found that SELENOK is required for imDC migration and phagocytosis.

## 1. Introduction

Dendritic cells (DCs) are proficient antigen-presenting cells that play an essential role in connecting innate and adaptive immune responses. In accordance with the levels of co-stimulatory molecules, such as the cluster of differentiation (CD)40 and CD80, DCs are divided into immature DCs (imDCs) and mature DCs (mDCs), which play an essential role in antigen phagocytosis and presentation, respectively [1]. Several previous studies have demonstrated that the immune function of chicken DCs was regulated by selenium [2,3]. Our recent studies have also illustrated that selenium can regulate the differentiation and maturation of human DCs [4,5], and its deficiency and excess impaired the DCs’ immune function [6] and reduced the number of splenic DCs in mice [7]. It is well known that selenium exerts its biological effect through selenoprotein, and the effect of selenium on the DCs’ immune function in chickens [2,3], humans [5] and mice [6,7] was related to changes in the levels of selenoprotein. It was found that selenium regulated the mRNA levels of selenoprotein K (SELENOK) in human imDCs and mDCs [5], and the protein levels involved in the regulation of DCs in mice [6]. We hypothesise that SELENOK may be involved in regulating the DCs’ immune function.

SELENOK is widely distributed in human and mouse tissues, with high expression levels in the immune system, and it is an endoplasmic reticulum (ER) transmembrane protein that regulates endoplasmic reticulum stress (ERS) and calcium (Ca^2+^) signalling [8]. Previous studies have shown that SELENOK deficiency reduces the proliferation and migration of T cells and neutrophils [9]. Furthermore, SELENOK is essential for Toll-like receptor-mediated activation [10] and FcγR-mediated phagocytosis [11] in macrophages. Recently, SELENOK was shown to be involved in the apoptosis of mouse neuronal cells [12] and the recovery of synaptic defects in Alzheimer’s disease model mice [13] through ERS or Ca^2+^ signalling. However, the role of SELENOK in ERS-induced alterations in the DCs’ immune function is still unclear. Numerous studies have shown that ERS can regulate the immune function of DCs, including cytokine production, DC maturation, antigen presentation and tumour immune response, by using an inositol-requiring enzyme 1 α/X-box-binding protein 1 (IRE1α/XBP1) signalling axis and PKR-like ER kinase (PERK) [14,15,16,17]. However, the role of activating transcription factor 4 (ATF4) and ATF6 in DCs is unclear. Therefore, we hypothesised that SELENOK could regulate ERS and, thus, affect the DCs’ immune function.

In this study, we evaluated the effect of SELENOK on the immune function of imDCs prepared from wild-type (WT) and SELENOK knockout (KO) mice. The effect of the ERS inducer, tunicamycin (Tm), on the migration, phagocytosis and phenotypic markers in WT imDCs was examined. Furthermore, ERS-induced migration and the phagocytosis of SELENOK-deficient imDCs were evaluated, and the ERS markers and phenotypic markers were detected.

## 2. Materials and Methods

### 2.1. Mice

Male WT mice (C57BL/6J) were purchased from the Guizhou Laboratory Animal Engineering Technology Centre, and the SELENOK knockout (KO) mice were obtained from the Saiye Biotechnology Co., Ltd. (Suzhou, China). The Institutional Animal Care and Use Committee at the Guizhou Medical University approved all the research protocols (No. 1800238).

### 2.2. Preparation of imDCs and mDCs

Fifty-one WT and twenty-four KO male mice aged eight weeks were used for the preparation of the imDCs and the mDCs, as previously described [6]. Briefly, bone marrow suspensions treated with erythrocyte lysate were cultured in RPMI 1640 medium containing foetal bovine serum (Hyclone, Logan, UT, USA), granulocyte-macrophage colony-stimulating factor (GM-CSF) and interleukin-4 (IL-4) (PeproTech, Rocky Hill, NJ, USA); the imDCs were obtained after 7 days. Lipopolysaccharides (Sigma–Aldrich, Darmstadt, Germany) were used to induce mDCs for another 2 days.

### 2.3. Cell Counting Kit-8 (CCK8) Assay

The imDCs and the mDCs prepared from the WT mice were treated with the ERS inducer, Tm (0.1, 0.5, 1, 2, 5, 10, 20 μg/mL) (Sigma–Aldrich, Darmstadt, Germany), for 24 h, and the control group was treated with dimethyl sulfoxide (DMSO) for the same time. Subsequently, a CCK8 assay was used to determine cell viability by using a microplate reader (Cytation5, Biotek, VT, USA) at 450 nm. For the SELENOK KO imDCs, the cells were treated with or without Tm (0.5 μg/mL), followed by a CCK8 assay. 

### 2.4. Migration Capability Detection

The free migration of the imDCs prepared from the WT mice was assayed by Transwell (Millipore, MA, USA) according to the previous description [6] after treatment with Tm (0.1, 0.5, 1 μg/mL) for 24 h. In addition, the imDCs were pre-treated with 30 μM Rhosin (MedChemExpress, Rocky Hill, NJ, USA), a RhoA inhibitor, for 24 h, followed by 1 μg/mL of Tm, and then the migration ability was detected. To evaluate the effect of SELENOK on the imDCs’ migration, the imDCs prepared from the KO mice were treated with or without Tm (0.5 μg/mL). Then, the migration ability was assayed by Transwell.

### 2.5. Phagocytosis Assay

Phagocytosis was assayed in terms of the cells’ ability to internalise FITC-dextran. The imDCs prepared from the WT mice were treated with Tm (0.1, 0.5, 1 μg/mL) for 24 h and co-cultured with FITC-dextran (1 mg/mL) (40 KDa, Sigma, Darmstadt, Germany) for 1.5 h, as previously described [6]. For the SELENOK KO imDCs, the cells were treated with or without Tm (0.5 μg/mL), followed by FITC-dextran treatment. The phagocytosis ability was then measured by flow cytometry at Ex./Em. = 488 nm/520 nm (NovoCyte, ACEA Biosciences, San Diego, CA, USA).

### 2.6. Calcium Measurement

Cytosolic Ca^2+^ levels in the WT and SELENOK-deficient imDCs treated with or without 0.5 μg/mL Tm were measured by flow cytometry [18]. After collecting the imDCs, they were incubated with 1 μM calcium fluorescent dye, Fluo-4 AM (Beyotime, Beijing, China), for 30 min at room temperature and then washed with phosphate buffer solution. Fluorescence intensity was detected by flow cytometry and the Ca^2+^ levels were monitored.

### 2.7. Real-Time PCR

The total RNA was isolated from the WT imDCs after treatment with Tm (0.1, 0.5, 1 μg/mL) for 24 h by the TRIzol method (Invitrogen, Carlsbad, CA, USA). The cDNA synthesis and RT-PCR were performed according to our previous description [6]. The primers used in this study are listed in Table 1. The ERS genes included the C/EBP homologous protein (CHOP), the binding immunoglobulin protein (BIP), ATF4 and ATF6. The reference gene was glyceraldehyde-3-phosphate dehydrogenase (GAPDH), and the mRNA levels of target genes were calculated using the 2^−ΔΔCT^ method [19].

### 2.8. Western Blot

The RIPA lysates were used to prepare protein extracts of DCs and Western blotting was performed. The antibodies RhoA (2117T), ATF4 (11815S), CHOP (5554T), BIP (3177T) and GAPDH (5174T) were purchased from CST; ATF6 (ab227830) and SELENOK (ab139949) were obtained from Abcam; SELENOS (15591-1-AP) was obtained from Proteintech; CD11c (GB11059) was obtained from Servicebio; CD40 (ER1803-54), CD80 (M1007-10) and CD205 (ET7107-58) were obtained from Huabio.

### 2.9. Statistical Analysis

The data were analysed by GraphPad Prism 8.0, and an analysis of variance (ANOVA) was applied to determine the statistical difference, followed by Tukey’s test. The data were presented as mean ± SD. The *p* values of less than 0.05 indicated statistical significance.

## 3. Results

### 3.1. ERS Resulted in Decreased DCs Cell Viability

**Figure 1:**
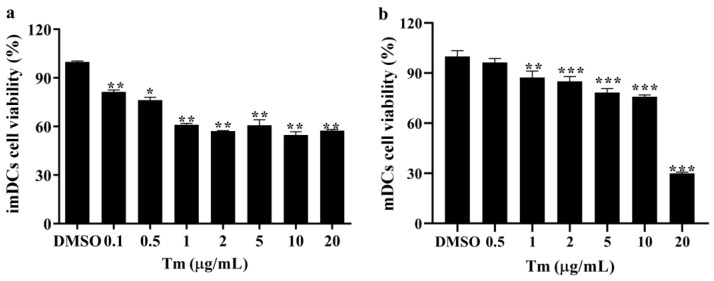
ERS resulted in decreased DC cell viability. The effect of the ERS inducer, tunicamycin (Tm), on the cell viability of imDCs (**a**) and mDCs (**b**) was determined by the CCK8 assay (*n* = 6). * *p* < 0.05, ** *p* < 0.01 and *** *p* < 0.001 were compared with DMSO-treated imDCs or mDCs. ERS, endoplasmic reticulum stress; DCs, dendritic cells; imDCs, immature dendritic cells; mDCs, mature dendritic cells; CCK8, Cell Counting Kit-8; DMSO, dimethyl sulfoxide.

The effect of the ERS inducer, Tm, on the cell viability of imDCs and mDCs was determined by the CCK8 assay. The results in Figure 1a show that the cell viability of imDCs was significantly reduced after Tm (0.1, 0.5, 1, 2, 5, 10, 20 μg/mL) treatment. Moreover, treatment with 0.5 μg/mL Tm did not affect mDCs cell viability, while the cell viability decreased significantly after treatment with 1, 2, 5, 10 or 20 μg/mL of Tm (Figure 1b).

### 3.2. ERS Decreased Migration and Increased Phagocytosis in imDCs

**Figure 2:**
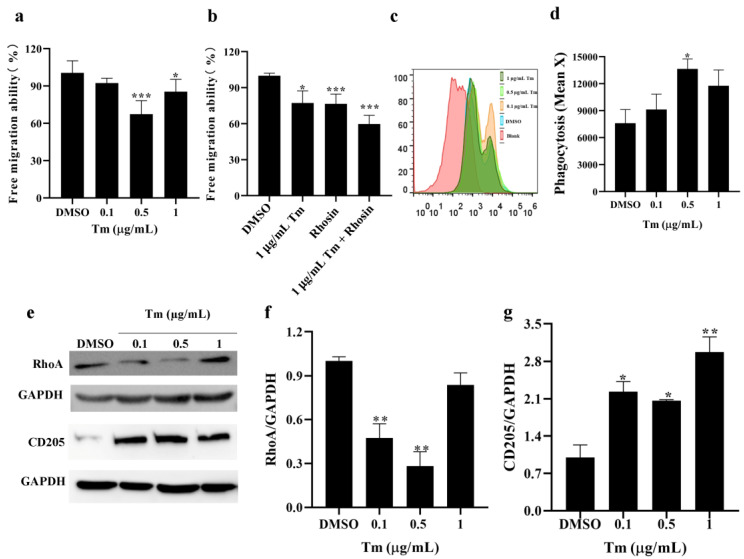
ERS decreased migration and increased phagocytosis in imDCs. (**a**) The free migration ability of imDCs treated with Tm (0.1, 0.5, 1 μg/mL) was assayed by Transwell (*n* = 3). (**b**) The free migration ability of imDCs treated with or without Rhosin, a RhoA inhibitor, under Tm treatment was assayed by Transwell (*n* = 3). (**c**,**d**) The phagocytosis ability in imDCs after treatment with Tm was measured by flow cytometry, and the fluorescence intensity was calculated (*n* = 3). (**e**–**g**) The protein levels of RhoA and CD205 in imDCs after treatment with Tm were assayed by Western blotting, and the density values were calculated (*n* = 3). * *p* < 0.05, ** *p* < 0.01 and *** *p* < 0.001 were compared with DMSO-treated imDCs. ERS, endoplasmic reticulum stress; imDCs, immature dendritic cells; Tm, tunicamycin; Ras homolog gene family member A, RhoA; CD205, cluster of differentiation 205; DMSO, dimethyl sulfoxide.

The migration and phagocytosis of imDCs treated with Tm were assayed by Transwell and flow cytometry, respectively. As shown in Figure 2a, the free migration of imDCs was reduced after treatment with 0.5 and 1 μg/mL of Tm, and Rhosin, a RhoA inhibitor, which could also lead to a decrease in the imDCs migration capacity (Figure 2b). In addition, Tm (0.1, 0.5 μg/mL) treatment downregulated the protein levels of RhoA (Figure 2e,f), indicating that a decrease in the RhoA protein level or activity is partly responsible for the decrease in Tm-induced imDC migration. The phagocytosis ability of imDCs was significantly enhanced by 0.5 μg/mL of Tm (Figure 2c,d), and elevated CD205 protein levels, an endocytic receptor of DC, were observed after the Tm (0.1, 0.5, 1 μg/mL) treatment (Figure 2e,g).

### 3.3. ERS Downregulated Phenotypic Markers in imDCs

**Figure 3:**
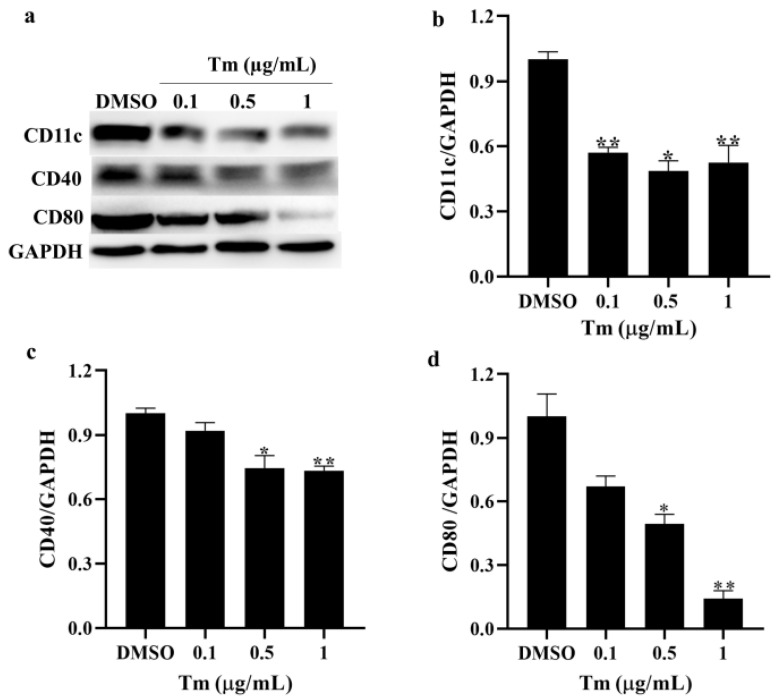
ERS downregulated the phenotypic markers in imDCs. (**a**–**d**) The protein levels of CD11c, CD40 and CD80 in imDCs after treatment with Tm were assayed by Western blotting, and the density values were calculated (*n* = 3). * *p* < 0.05 and ** *p* < 0.01 were compared with DMSO-treated imDCs. ERS, endoplasmic reticulum stress; imDCs, immature dendritic cells; CD11c, cluster of differentiation 11c; CD40, cluster of differentiation 40; CD80, cluster of differentiation 80; Tm, tunicamycin; DMSO, dimethyl sulfoxide.

The phenotypic markers of the imDCs treated with Tm were measured by Western blotting. It was shown that the phenotypic markers CD11c, CD40 and CD80 were downregulated (Figure 3), suggesting that Tm induces a decrease in the total protein levels of imDC phenotypic markers, thereby affecting their function.

### 3.4. ERS Increased the Levels of ERS Markers in imDCs

**Figure 4:**
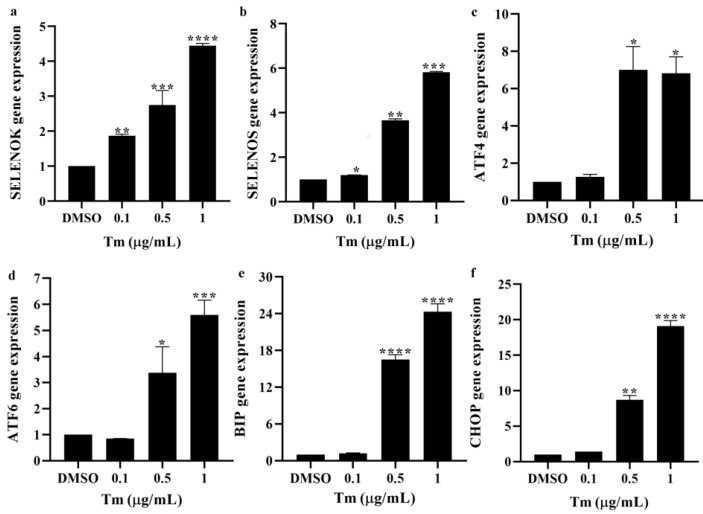
ERS increased the mRNA levels of the ERS markers in imDCs. (**a**,**b**) The mRNA levels of SELENOK and SELENOS, as well as (**c**–**f**) the ERS markers ATF4, ATF6, BIP and CHOP in imDCs after treatment with Tm, were assayed by RT-PCR (*n* = 3). * *p* < 0.05, ** *p* < 0.01, *** *p* < 0.001 and **** *p* < 0.0001 were compared with DMSO-treated imDCs. ERS, endoplasmic reticulum stress; imDCs, immature dendritic cells; SELENOK, selenoprotein K; SELENOS, selenoprotein S; ATF4, activating transcription factor 4; ATF6, activating transcription factor 6; BIP, binding immunoglobulin protein; CHOP, C/EBP homologous protein; Tm, tunicamycin; DMSO, dimethyl sulfoxide.

**Figure 5:**
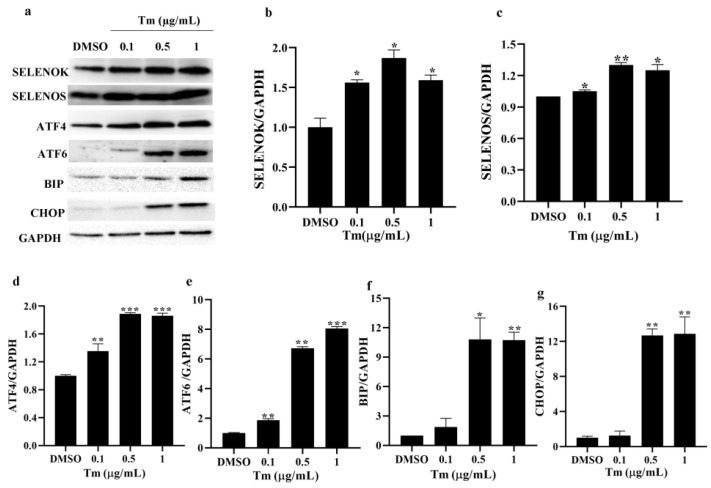
ERS increased the protein levels of the ERS markers in imDCs. (**a**–**c**) The protein levels of SELENOK and SELENOS, as well as (**a**,**d**–**g**) the ERS markers ATF4, ATF6, BIP and CHOP in imDCs after treatment with Tm, were assayed by Western blotting, and the density values were calculated (*n* = 3). * *p* < 0.05, ** *p* < 0.01 and *** *p* < 0.001 were compared with DMSO-treated imDCs. ERS, endoplasmic reticulum stress; imDCs, immature dendritic cells; SELENOK, selenoprotein K; SELENOS, selenoprotein S; ATF4, activating transcription factor 4; ATF6, activating transcription factor 6; BIP, binding immunoglobulin protein; CHOP, C/EBP homologous protein; Tm, tunicamycin; DMSO, dimethyl sulfoxide.

The mRNA and protein levels of the ERS markers SELENOK and SELENOS were assayed by RT-PCR and Western blotting, respectively. The results showed that the gene (Figure 4) and protein (Figure 5) levels of SELENOK, SELENOS, ATF4, ATF6, BIP and CHOP were gradually increased in Tm-treated imDCs, suggesting that altered immune function was due to an alteration in the imDCs markers, which is associated with increased ERS levels, and SELENOK and SELENOS were involved in the regulation of ERS.

### 3.5. SELENOK Deficiency Induced Increased Migration and Decreased Phagocytosis in imDCs

**Figure 6:**
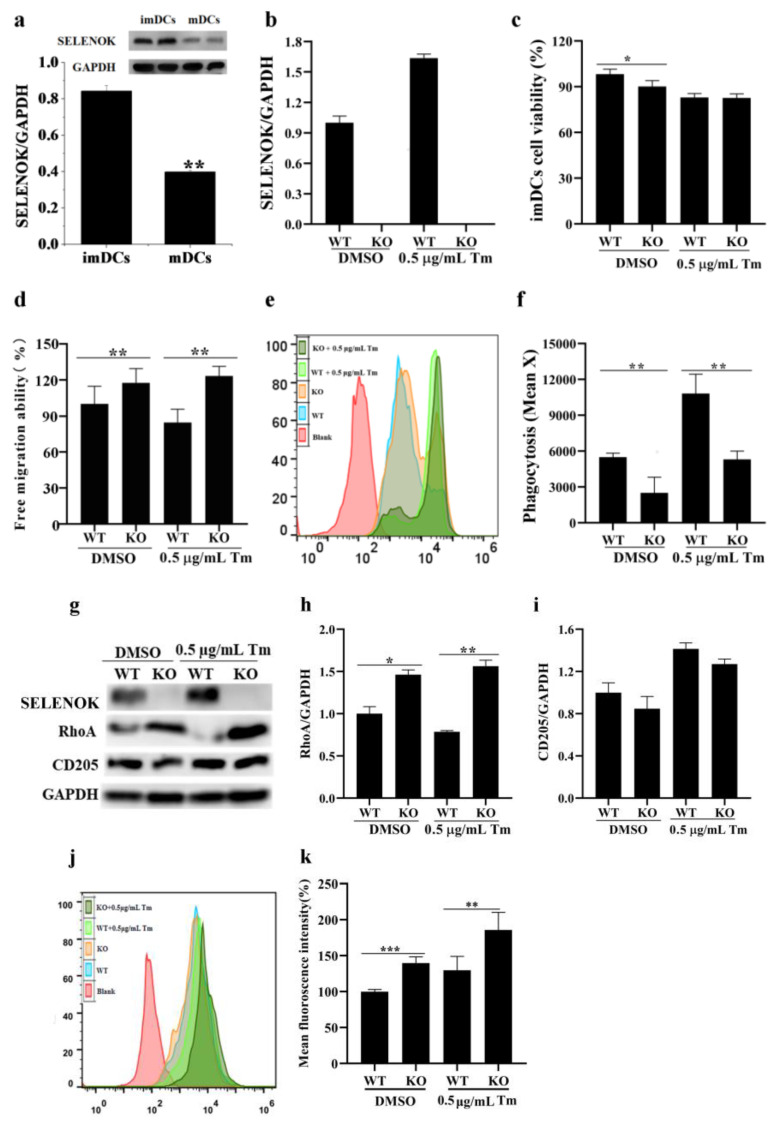
SELENOK deficiency induced increased migration and decreased phagocytosis in imDCs. (**a**) The protein levels of SELENOK in imDCs and mDCs were assayed by Western blotting, and the density values were calculated (*n* = 3). (**b**) The density value analysis of SELENOK KO validation in imDCs after treatment with Tm (*n* = 3). (**c**) Effect of SELENOK KO on the viability of imDCs after treatment with Tm (*n* = 3). (**d**) Effect of SELENOK KO on the migration of imDCs after treatment with Tm (*n* = 3). (**e**,**f**) Effect of SELENOK KO on the phagocytosis ability of imDCs after treatment with Tm (*n* = 3). (**g**–**i**) Effect of SELENOK KO on the protein levels of RhoA and CD205 in imDCs after treatment with Tm (*n* = 3). (**j**,**k**) Effect of SELENOK KO on the levels of Ca^2+^ in imDCs after treatment with Tm (*n* = 3). * *p* < 0.05, ** *p* < 0.01 and *** *p* < 0.001 were compared with DMSO- or Tm-treated WT imDCs. SELENOK, selenoprotein K; imDCs, immature dendritic cells; mDCs, mature dendritic cells; Tm, tunicamycin; RhoA, Ras homolog gene family member A; CD205, cluster of differentiation 205; DMSO, dimethyl sulfoxide; WT, wild-type; KO, knockout.

The results in Figure 6a indicate that SELENOK protein levels were downregulated in mDCs when compared to imDCs, and SELENOK deficiency in imDCs of SELENOK KO mice was observed (Figure 6b,g). SELENOK-deficient imDCs showed reduced viability (Figure 6c), increased migratory capacity (Figure 6d) and reduced antigen phagocytosis (Figure 6e,f). Compared with Tm-treated WT imDCs, Tm-treated SELENOK-deficient imDCs showed unchanged cell viability (Figure 6c), increased migratory capacity (Figure 6d) and decreased antigen phagocytosis (Figure 6e,f); changes in migration capacity were RhoA-dependent (Figure 6d,g,h). Compared with WT and KO controls, Tm treatment showed increased phagocytosis and CD205. Still, the decrease in the phagocytosis induced by SELENOK deficiency did not lead to significant downregulation of CD205 (Figure 6e–g,i). Furthermore, SELENOK deficiency led to elevated Ca^2+^ levels with or without Tm treatment (Figure 6j,k).

### 3.6. SELENOK Deficiency-Induced SELENOS Upregulation Was Involved in ERS

**Figure 7:**
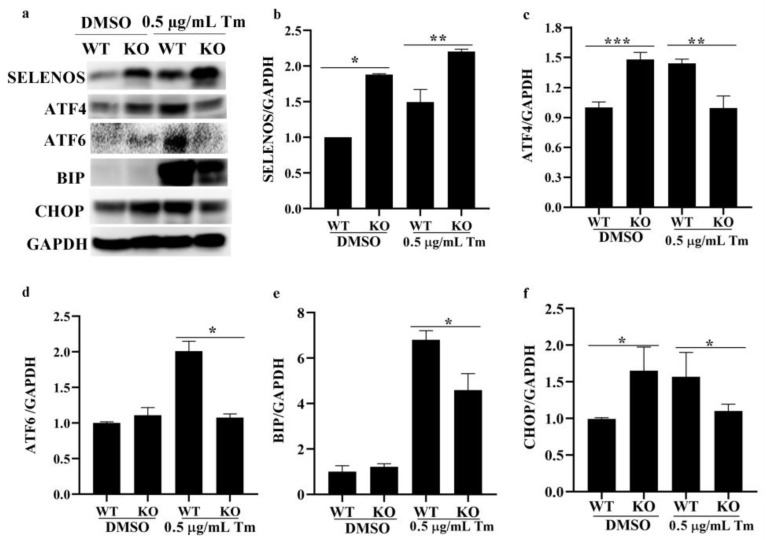
SELENOK deficiency-induced SELENOS upregulation was involved in ERS in imDCs. (**a**–**f**) The protein levels of SELENOS and the ERS markers ATF4, ATF6, BIP and CHOP in WT and KO imDCs after treatment with Tm were assayed by Western blotting, and the density values were calculated (*n* = 3). * *p* < 0.05, ** *p* < 0.01 and *** *p* < 0.001 were compared with DMSO- or Tm-treated WT imDCs. SELENOK, selenoprotein K; SELENOS, selenoprotein S; ERS, endoplasmic reticulum stress; imDCs, immature dendritic cells; ATF4, activating transcription factor 4; ATF6, activating transcription factor 6; BIP, binding immunoglobulin protein; CHOP, C/EBP homologous protein; Tm, tunicamycin; DMSO, dimethyl sulfoxide; WT, wild-type; KO, knockout.

As shown in Figure 7, SELENOK deficiency upregulated the expression of SELENOS, ATF4 and CHOP in control imDCs. In Tm-treated imDCs, SELENOK deletion induced significant upregulation of SELENOS and significant downregulation of ATF4, ATF6, BIP and CHOP, indicating that SELENOK deficiency-induced SELENOS upregulation is involved in the regulation of ERS.

### 3.7. SELENOK Deficiency Downregulated Phenotypic Markers in imDCs

**Figure 8:**
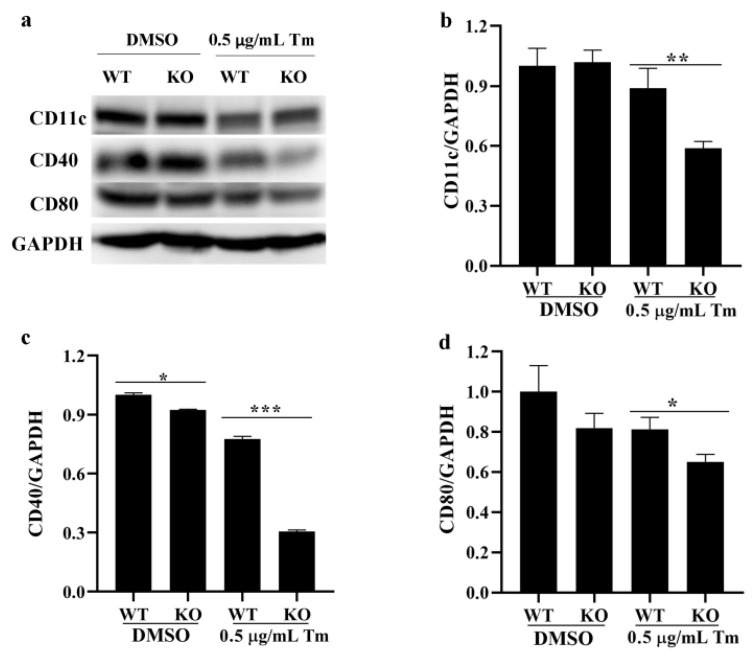
SELENOK deficiency downregulated the phenotypic markers in imDCs. (**a**–**d**) The protein levels of CD11c, CD40 and CD80 in WT and KO imDCs after treatment with Tm were assayed by Western blotting, and the density values were calculated (*n* = 3). * *p* < 0.05, ** *p* < 0.01 and *** *p* < 0.001 were compared with DMSO- or Tm-treated WT imDCs. SELENOK, selenoprotein K; imDCs, immature dendritic cells; CD11c, cluster of differentiation 11c; CD40, cluster of differentiation 40; CD80, cluster of differentiation 80; Tm, tunicamycin; DMSO, dimethyl sulfoxide; WT, wild-type; KO, knockout.

It was shown that SELENOK deficiency reduced the expression of CD40, and the expression of CD11c, CD40 and CD80 was further reduced in Tm-treated SELENOK-deficient imDCs compared to Tm-treated WT imDCs (Figure 8), suggesting that SELENOK deficiency can downregulate the phenotypic markers of imDCs and affect their immune function.

## 4. Discussion

Our study showed that the ERS inducer, Tm, can downregulate the phenotypic markers in WT imDCs through ERS, leading to decreased migration and increased phagocytosis. Furthermore, SELENOK deficiency downregulated the phenotypic markers in imDCs by SELENOS-regulated ERS, which led to increased migration and decreased phagocytosis.

**Figure 9:**
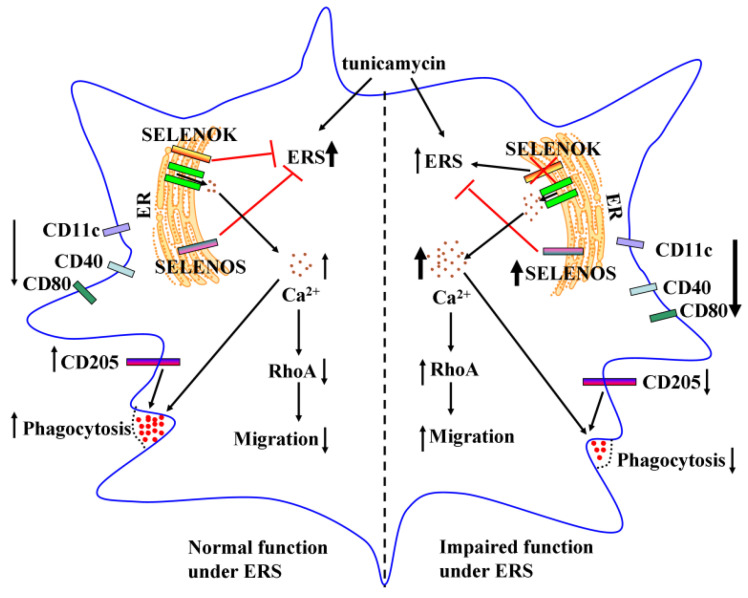
Schematic diagram of the mechanism of imDCs dysfunction mediated by SELENOK. Under ERS conditions, SELENOK, SELENOS and ERS markers were upregulated, and the phenotypic markers were downregulated. The downregulation of RhoA inhibited migration and increased Ca^2+^ or CD205 enhanced phagocytosis in WT imDCs. In SELENOK KO imDCs, SELENOS upregulation attenuated ERS levels, decreased phenotypic markers, upregulated RhoA due to increased Ca^2+^, which promoted migration, increased Ca^2+^ or decreased CD205, which inhibited phagocytosis. imDCs, immature dendritic cells; SELENOK, selenoprotein K; ERS, endoplasmic reticulum stress; SELENOS, selenoprotein S; RhoA, Ras homolog gene family member A; CD205, cluster of differentiation 205; WT, wild-type; KO, knockout.

Several previous studies suggested that SELENOK plays an essential role in the immune function of T cells, neutrophils and macrophages [9,10,11]. However, how SELENOK regulates the function of DCs is still unknown. In the present study, SELENOK deficiency downregulated the phenotypic markers of imDCs under ERS conditions. It modulated RhoA-dependent migration and Ca^2+^/CD205-mediated phagocytosis (Figure 9), indicating that SELENOK is essential for imDCs’ migration and phagocytosis.

It was found that reduced SELENOK levels increased ERS-induced apoptosis in the HepG2 cells [22]. The deficiency of SELENOK increased West Nile virus-induced death in mice [9] and induced apoptosis in mice neurons [12]. Overexpression of SELENOK could decrease the viability and increase apoptosis in the BGC-823 cells, but did not affect the HEK-293 cells [23]. In this study, the cell viability of imDCs and mDCs was decreased after the ERS inducer treatment (Figure 1), and SELENOK and SELENOS were upregulated by Tm in imDCs (Figure 4 and Figure 5). In addition, SELENOK deficiency induced a decrease in imDCs cell viability, but had no changes after Tm treatment (Figure 6), which was related to the increase in SELENOS (Figure 7). It was shown that SELENOK [9,12,22] and SELENOS [24,25] have a protective role in ERS-induced cell death or apoptosis. In addition, SELENOK and SELENOS can form a complex involved in regulating ERS [26], which is consistent with the Tm-induced upregulation of SELENOK and SELENOS.

SELENOK deficiency inhibits the migration of human melanoma cells by reducing the Ca^2+^ flux [27]. Still, overexpression of SELENOK inhibits the migration of human gastric cancer cells by increasing Ca^2+^ levels [23]. It reduces the migration of choriocarcinoma cells by ERK, p38 MAPK and Akt pathways [28], suggesting that SELENOK has different effects on the migration of different tumours. For immune cells, the migration of T cells and neutrophils and the migration and phagocytosis of macrophages in SELENOK-deficient mice were decreased via deficiency in Ca^2+^ flux [9,29]. Furthermore, SELENOK overexpression enhances microglia migration and phagocytosis through an inositol triphosphate receptor (IP3R)-induced increase in Ca^2+^ levels [30]. In this study, we found that SELENOK deficiency induced an increase in cytosolic Ca^2+^ levels, resulting in increased migration and decreased phagocytosis. In addition, changes in the migration due to Ca^2+^ levels were RhoA-dependent, and Ca^2+^ or CD205 were associated with the regulation of phagocytosis in imDCs (Figure 2 and Figure 6). Our previous study also revealed that a decrease in RhoA led to a decrease in imDCs migration [6]. Previous reports have also shown that increased Ca^2+^-dependent RhoA could lead to increased migration in intestinal epithelial cells [31]. This study consistently showed that increased Ca^2+^ levels increased RhoA-mediated migration in SELENOK-deficient imDCs. Recently, human CD205 has not been predicted to bind to Ca^2+^, which contributes to oligosaccharide binding to other endocytic receptors [32,33], suggesting that Ca^2+^, CD205 or other endocytic receptors are jointly involved in the phagocytosis of imDCs. In addition, mechanical stiffness-induced PIEZO1-mediated increases in Ca^2+^ levels promoted the antitumor response in DCs [18]. In contrast, the increase in Ca^2+^ levels inhibited antigen phagocytosis of imDCs in this study, which is probably due to the developmental state of DCs and different treatment conditions.

An increase in misfolded proteins and reactive oxygen species or disruptions in Ca^2+^ homeostasis induce ERS, leading to unfolded protein reactions that include the IRE1, PERK and ATF6 pathways [12,34]. ERS plays an essential role in the immune response of T cells, B cells, macrophages and DCs [34]. Reports showed that IRE1/XBP1 and PERK/CHOP could affect maturation, antigen presentation, cytokine secretion and antitumor response in DCs [14,15,16,17,34,35,36,37,38,39]. In this study, the ERS markers ATF4, ATF6, BIP and CHOP were increased after treatment with Tm in WT imDCs, leading to decreased migration and enhanced phagocytosis (Figure 2, Figure 3, Figure 4 and Figure 5). In contrast, in SELENOK-deficient imDCs, SELENOS upregulation diminished ERS levels, leading to increased migration and decreased phagocytosis (Figure 6, Figure 7 and Figure 8). However, impaired ER function resulted in a decrease in the phenotypic markers of the WT or KO imDCs. Studies have shown that ERS in the tumour microenvironment impairs the antitumor immune response of DCs [36,37,38]. Combined with the results in this study, we suggest that SELENOK-deficient DCs in tumour tissues would attenuate their antitumor immune response. The human and mouse SELENOK proteins have high sequence homology [8], and we hypothesise that SELENOK performs the same role in human DCs, but further experimental verification is required.

## 5. Conclusions

In conclusion, this study found that SELENOK is required for imDC migration and phagocytosis. SELENOK has essential functions in tumour cell invasion and migration [23,27,28], and the migration and phagocytosis of immune cells [9,29,30]. SELENOK is associated with the prognosis of lung adenocarcinoma and melanoma [8,27,40] and can be a potential target for tumour immunotherapy. Previous studies have only focused on the effect of SELENOK on tumour cells or immune cell function. We anticipate that it is essential to investigate the effect of SELENOK on immune cell infiltration in the tumour microenvironment and on tumour cell–immune cell interactions. Importantly, SELENOK, in addition to regulating ERS, is a palmitoylation-modifying protease. It can promote DHHC6-catalysed palmitoylation of CD36, which is required for macrophage function [8,29,41,42]. In addition, SELENOK plays an important role in the differentiation of myogenic cells mediated by satellite cells [43], and the degradation of SELENOK is required for adipocyte differentiation [44]. The effects of SELENOK-mediated palmitoylation on DCs’ function and SELENOK deficiency on DCs’ differentiation and maturation are worthy of future investigation.

## Figures and Tables

**Table 1:** Real-time PCR primers.

Symbol	Accession Number	Forward Primer	Reverse Primer	Refs.
GAPDH	NM_001289726	tgacatcaagaaggtggtgaagc	ccctgttgctgtagccgtattc	[20]
SELENOK	NM_019979	tccacgaagaatgggtagga	gcttctcagagcagacatttacct	[20]
SELENOS	NM_024439	cagaagattgaaatgtgggacagc	cctttggggatgacagatgaagtag	[20]
CHOP	NM_007837	ccaacagaggtcacacgcac	tgactggaatctggagagcga	[21]
BIP	NM_001163434	tgtggtacccaccaagaagtc	ttcagctgtcactcggagaat	[21]
ATF4	NM-009716	cgagatgagcttcctgaacagc	ggaaaaggcatcctccttgc	[21]
ATF6	NM_001081304	taccacccacaacaagacca	tgatgatcccggagataagg	[21]

## Data Availability

Data are contained within the article.
